# *ACE2* and *TMPRSS2* variation in savanna monkeys (*Chlorocebus* spp.): Potential risk for zoonotic/anthroponotic transmission of SARS-CoV-2 and a potential model for functional studies

**DOI:** 10.1371/journal.pone.0235106

**Published:** 2020-06-23

**Authors:** Christopher A. Schmitt, Christina M. Bergey, Anna J. Jasinska, Vasily Ramensky, Felicity Burt, Hannes Svardal, Matthew J. Jorgensen, Nelson B. Freimer, J. Paul Grobler, Trudy R. Turner

**Affiliations:** 1 Department of Anthropology, Boston University, Boston, Massachusetts, United States of America; 2 Department of Genetics, Rutgers University, New Brunswick, New Jersey, United States of America; 3 Center for Neurobehavioral Genetics, Semel Institute for Neuroscience and Human Behavior,University of California—Los Angeles, Los Angeles, California, United States of America; 4 Institute of Bioorganic Chemistry, Polish Academy of Sciences, Poznan, Poland; 5 Eye on Primates, Los Angeles, California, United States of America; 6 Federal State Institution “National Medical Research Center for Therapy and Preventive Medicine” of the Ministry of Healthcare of the Russian Federation, Moscow, Russia; 7 Division of Medical Virology, National Health Laboratory Service, Bloemfontein, Free State, South Africa; 8 Division of Virology, Faculty of Health Sciences, University of the Free State, Bloemfontein, Free State, South Africa; 9 Department of Biology, University of Antwerp, Antwerp, Belgium; 10 Naturalis Biodiversity Center, Leiden, The Netherlands; 11 Department of Pathology, Section on Comparative Medicine, Wake Forest School of Medicine, Winston-Salem, North Carolina, United States of America; 12 Department of Genetics, University of the Free State, Bloemfontein, Free State, South Africa; 13 Department of Anthropology, University of Wisconsin–Milwaukee, Milwaukee, Wisconsin, United States of America; Tulane University, UNITED STATES

## Abstract

The COVID-19 pandemic, caused by the coronavirus SARS-CoV-2, has devastated health infrastructure around the world. Both ACE2 (an entry receptor) and TMPRSS2 (used by the virus for spike protein priming) are key proteins to SARS-CoV-2 cell entry, enabling progression to COVID-19 in humans. Comparative genomic research into critical ACE2 binding sites, associated with the spike receptor binding domain, has suggested that African and Asian primates may also be susceptible to disease from SARS-CoV-2 infection. Savanna monkeys (*Chlorocebus* spp.) are a widespread non-human primate with well-established potential as a bi-directional zoonotic/anthroponotic agent due to high levels of human interaction throughout their range in sub-Saharan Africa and the Caribbean. To characterize potential functional variation in savanna monkey *ACE2* and *TMPRSS2*, we inspected recently published genomic data from 245 savanna monkeys, including 163 wild monkeys from Africa and the Caribbean and 82 captive monkeys from the Vervet Research Colony (VRC). We found several missense variants. One missense variant in *ACE2* (X:14,077,550; Asp30Gly), common in *Ch*. *sabaeus*, causes a change in amino acid residue that has been inferred to reduce binding efficiency of SARS-CoV-2, suggesting potentially reduced susceptibility. The remaining populations appear as susceptible as humans, based on these criteria for receptor usage. All missense variants observed in wild *Ch*. *sabaeus* populations are also present in the VRC, along with two splice acceptor variants (at X:14,065,076) not observed in the wild sample that are potentially disruptive to ACE2 function. The presence of these variants in the VRC suggests a promising model for SARS-CoV-2 infection and vaccine and therapy development. In keeping with a One Health approach, characterizing actual susceptibility and potential for bi-directional zoonotic/anthroponotic transfer in savanna monkey populations may be an important consideration for controlling COVID-19 epidemics in communities with frequent human/non-human primate interactions that, in many cases, may have limited health infrastructure.

## Introduction

The emergence of the severe acute respiratory syndrome coronavirus 2 (SARS-CoV-2) in China in December 2019 has since led to a devastating human pandemic of COVID-19 [1], the potentially fatal respiratory disease resulting from infection with the novel coronavirus [2]. Due to the ongoing severity of the pandemic in humans, little attention has yet focused on human/animal transmission beyond the initial presumed zoonotic event with a bat and/or pangolin host [3]. Although potential anthroponotic transmissions to domesticated animals has been detected, these transmissions have been relatively rare (e.g., ferrets, cats, dogs) [4] and human-to-human transmission is the primary risk [5] [6]. Aspects of the biology of SARS-CoV-2 infection call for stricter scrutiny of such transmissions among non-human primates kept as pets, among wild non-human primates, and other populations that may have frequent exposure to potentially infected humans.

The angiotensin-converting enzyme 2 (ACE2), previously identified as the key receptor for the SARS coronavirus [7], has since also been identified as one of the key binding receptors by which SARS-CoV-2 gains entry to cells [8] [9] [10]. The spike protein of SARS-CoV-2 contains a variable receptor binding domain (RBD) which binds to the ACE2 receptor facilitating entry of the virus into cells. Mutations at critical sites in the RBD appear to alter the binding affinity to ACE2 receptors, facilitating spill-over events into other species. SARS-CoV-2 likely originated in bats with zoonotic spill-over, via an intermediate host, to humans [3] [11]. In the same way that mutations in the spike protein have altered binding efficacy to the ACE2 receptor, evidence suggests that mutations or natural variants in the ACE2 receptor may influence susceptibility or resistance of a species to infection. Identifying key receptors associated with infectivity and transmission will be important for understanding host range and potentially susceptible hosts. It has been shown that changes in key residues of ACE2 influence the receptor efficiency and consequently entry of severe acute respiratory syndrome coronavirus 1 (SARS-CoV) into cells [12]. To gain insight into host range for SARS-CoV-2 based on receptor usage, alignment of predicted amino acid sequences for human, non-human primates and selected domestic animals showed shared identity at binding regions and critical residues [13].

Recent research has supported the idea that, due to ACE2 receptor congruence–particularly at critical binding sites for the SARS-CoV-2 spike receptor binding domain–catarrhine primates may be as susceptible to infection with COVID-19-like disease as humans [13] [14] [15], presenting the possibility of bi-directional human-non-human primate infections in areas with human-wildlife contact. Although rhesus macaques (*Macaca mulatta*) have been experimentally infected and do show COVID-19-like symptoms upon infection [16] [17] [18] zoonotic/anthroponotic potential in wild populations has not yet been investigated. This is cause for great concern, not just from a global and community health perspective, but also from a conservation perspective, as many critically endangered non-human primates may also be vulnerable to COVID-19 [14].

Cell entry is facilitated by binding of SARS-CoV-2 to ACE2 receptors and on spike protein priming by host cell proteases. Transmembrane serine protease 2 (TMPRSS2) is a cellular protease that had previously been shown to be a proteolytic activator of both SARS-CoV and Middle East respiratory syndrome coronavirus (MERS-CoV), responsible for cleaving the spike protein of each into a conformation that facilitates cell membrane fusion and increases infectivity [19] [20]. TMPRSS2 also cleaves the ACE2 binding site itself, which further increases coronavirus infectivity [21]. It has since been shown to similarly prime SARS-CoV-2 for infection [22]. As in SARS-CoV [20], a TMPRSS2 inhibitor has been shown to block SARS-CoV-2 from cellular entry, and may serve as a treatment for COVID-19 [10]. This suggests that key polymorphisms in TMPRSS2 protein structure might also yield differential susceptibility to infection with SARS-CoV-2. This gene region shows pleiotropy in primates, and has previously shown signs of positive selection across the order while investigating its putative role in influencing the properties of seminal fluid [23]. Androgens regulate expression of *TMPRSS2*, and its functionality is autocatalyzed by cleavage of the protein at the R255 residue, yielding the functional form [24]. Previous research has identified the proteolytic activation function of TMPRSS2 to be dependent on the coordination of a catalytic triad comprised of the H296, D345, and S441 residues [20].

Savanna monkeys (*Chlorocebus* spp.) are a catarrhine primate that is ubiquitous throughout sub-Saharan African savanna environments [25]. They are often characterized as problem animals within this range due to their easy co-habitation with human settlements ranging from rural farmland to densely packed urban areas [26] [27] [28]. Green monkeys (*Ch*. *sabaeus*) are also endemic to the Caribbean islands of St. Kitts, Nevis, and Barbados, having been brought to the islands over 300 years ago [29], where they are densely populated and have frequent contact with humans [27]. The established Caribbean populations have since spread to the islands of St. Martin/St. Maarten and Tortola as escaped pets [30]. There is also a small feral population of green monkeys established near Ft. Lauderdale, Florida, in the United States [31].

Savanna monkeys have been shown to be more susceptible than rhesus macaques to respiratory infection and viral replication when exposed to coronavirus SARS-CoV, but when infected they show no evident external symptoms of disease [32]. Green monkeys (*Ch*. *sabaeus*) exposed to SARS-CoV (Urbani strain) via intratracheal swab have high titers of the virus in tracheal and lung tissue accompanied by pneumonitis on days 2 through 4 of infection, followed by eventual resolution [32]. Given the increased severity of response to SARS-CoV in savanna monkeys compared to rhesus, recent evidence that rhesus macaques show COVID-19-like symptoms upon SARS-CoV-2 infection [16] [17] [18] strongly suggests that savannah monkeys will also respond to infection with COVID-19-like disease. This not only raises the potential of the savanna monkey as a potential model for developing vaccines and therapeutics against COVID-19, but also for anthroponotic and zoonotic infection where savanna monkeys and humans meet.

Contact between savanna monkeys and humans with transmissible COVID-19 infections seem an inevitability, even with social distancing and stay-at-home orders in place in many African and Caribbean nations showing high infection rates. This is primarily due to their frequent raiding of crops and interactions with human refuse and other potential fomites in the wild [26] [27] [33]. Use of human resources as ‘hotspots’ of interest could potentially exacerbate animal disease propagation in human-frequented areas [34]. Urban areas present a special risk, as urban savanna monkeys are more habituated to human proximity and less likely to flee when humans are near [35]. Indeed, several zoonoses and anthroponoses have already been documented to pass between humans and wild savanna monkeys throughout their range (e.g., simian foamy virus [36], *Staphylococcus aureus* [37], gastrointestinal parasites [38]). The abundance of sanctuary and rehabilitation centers that focus on caring for those savanna monkeys that have been adversely affected by human impacts serves as another key mediator of potential transmission [39] [40] [41].

Given the high zoonotic/anthroponotic transmission potential between savanna monkeys and humans, we used recently published genome sequence data from 163 wild savanna monkeys [42] and 82 captive green monkeys from the Vervet Research Colony (VRC) at Wake Forest School of Medicine [43] to document variation in the *ACE2* and *TMPRSS2* gene regions in this widespread genus. Our goals were 1) to assess the likelihood of anthroponotic infection of wild savanna monkeys upon exposure to infected humans, in order to evaluate the risk of savanna monkey populations becoming 2a) as highly impacted as human populations by potential epidemics, 2b) vectors of infection to humans via zoonotic exposure and 2c) potential reservoirs of infection with potential for the post-pandemic initiation of novel COVID-19 outbreaks among humans; 3) identify natural genetic models for functional studies of the host proteins critical to SARS-CoV-2 entry in the context of transmission of the virus and development of treatments for COVID-19; and 4) assess if captive savanna monkey colonies might be appropriate for modeling COVID-19 disease infection and therapeutics studies, as with SARS-CoV.

## Materials and methods

Whole genome sequence data (median 4.4X coverage, with at least one member per taxon sequenced to at least 10X coverage) was previously generated for 163 individual wild savanna monkeys as part of research conducted by the International Vervet Research Consortium (for details, see [42] [44]. Raw sequence data were aligned to the Vervet-AGM reference assembly (ChlSab1.1 [45]), and variant calls are archived and publicly available online via *Ensembl* (Release 99 [46]). These data include resequencing from populations across sub-Saharan Africa and the Caribbean, including: 16 grivets (*Ch*. *aethiops*) from Ethiopia; 14 vervets (C*h*. *pygerythrus hilgerti*) from Kenya and Tanzania; 16 malbroucks (*Ch*. *cynosuros*) from Zambia; 51 vervets (*Ch*. *pygerythrus pygerythrus*) from South Africa and Botswana; 11 tantalus monkeys (*Ch*. *tantalus*) from the Central African Republic; and 63 African green monkeys (*Ch*. *sabaeus*), 22 from The Gambia, 2 from Ghana, 34 from St. Kitts & Nevis, and 5 from Barbados [42]. Although the taxonomy of these populations is contentious and currently unresolved [25], for simplicity we have here chosen to follow the taxonomic designations of Groves [47]. Variant calls in in the *ACE2* (X:14,030,233–14,077,785) and *TMPRSS2* (2:85362117–85412926) gene regions in wild savanna monkeys were inspected using the Variant Table function, and filtered by annotations produced by Ensembl genebuild [48].

Allele frequencies in the VRC monkeys are reported here for 82 monkeys with moderate to high sequencing coverage depth (10-30X). Variant discovery and genotype calling steps are described in detail by Ramensky et al. [43]. For variant annotation, we used Ensembl Variant Effect Predictor [49]. Sequenced monkeys in the VRC are highly related, so in this case MAF should be interpreted as alternative allele occurrence rather than frequency in the strict statistical sense, which implies independent observations.

Finally, we used SSIPe [50] to assess the change in protein binding affinity caused by the discovered Asp30Gly mutation at the critical residue identified via alanine scanning mutagenesis analyses performed by Melin et al. [14]. The change in free energy due to the mutation was inferred by comparing against a reference interaction of human ACE2 receptor bound to the SARS-CoV-2 spike receptor binding domain (PBD 6M0J).

## Results

There is substantial variation in the *ACE2* sequence of wild savanna monkeys. None of the detected variants are predicted to cause frameshifts or premature stops in the transcription process. Exonic variants include 6 missense variants, 14 synonymous variants, and two potential canonical splice region variants (Table 1), which are the only examples of putative protein-truncating variants in the vervet *ACE2* discovered so far. Other detected variants include 43 in the 3’ UTR region, and 680 intronic variants including 3 potential splice region variants. Missense variants in savanna monkeys appear to occur only in the Ethiopian *Ch*. *aethiops* and West African/Caribbean *Ch*. *sabaeus* populations sampled. Only one of the missense-mediated changes in amino acid residues, also in *Ch*. *sabaeus* (X:14,077,550; Asp30Gly), involves a conversion at one of the critical binding sites for SARS-CoV-2 identified by Melin et al. [14]. This substitution is predicted to destabilize the binding of SARS-CoV-2 to ACE2 receptors (ΔΔG_bind_ = 0.958 kcal/mol), and of all the point mutations analyzed in a recent survey of primates [14], Asp30Gly reduces the predicted binding affinity more than all but the Tyr21His variant, which is found in most strepsirrhines (lemurs and lorisoids) and all platyrrhines (monkeys from the Americas) considered.

**Table 1:** Potential functional variants in *ACE2* gene region sequence among wild savanna monkeys.

Position	Variant	Consequence	AAF^a^	AA	Pos_AA_	Notes
X:14035311	T/C	Missense	0.09	I/V	753	Alt. allele prevalent in Ethiopia (AAF = 0.94); absent in all others
X:14035353*	C/T	Missense	0.33	V/I	739	Alt. allele only in *Ch*. *sabaeus* (AAF = 0.75–1; AAF_VRC_ = 0.84).
X:14035354	G/A	Synonymous	0.09	P	738	Alt. allele prevalent in Ethiopia (AAF = 0.94); absent in all others
X:14035357	G/A	Synonymous	0.02	S	737	Alt. allele only found in Ethiopia (AAF = 0.16)
X:14035374*	T/C	Missense	0.97	I/V	732	Ref. allele only found in St. Kitts (AAF = 0.75; AAF_VRC_ = 0.85).
X:14041898	T/C	Synonymous	0.13	G	629	Alt. allele only found in southern Africa (AAF = 0.36–1.00)
X:14043283	G/C	Synonymous	0.04	L	585	Alt. allele predominantly found in Central African Republic (AAF = 0.36)
X:14043289*	T/G	Synonymous	0.06	P	583	AAF_VRC_ = 0.01
X:14043304	G/A	Synonymous	0.03	N	578	Alt. allele only found in South Africa (AAF = 0.10)
X:14043773	T/A	Intronic SRV	0.45	-	-	Fixation of minor allele in *Ch*. *sabaeus*
X:14043797	T/A	Missense	0.02	E/V	549	Alt. allele only found in Ethiopia (AAF = 0.19)
X:14045023*	C/G	Missense	0.62	E/D	483	Ref. allele near fixation in *Ch*. *sabeaus* (AAF = 0.00–0.50; AAF_VRC_ = 0.01).
X:14049928	G/A	Synonymous	0.01	I	358	
X:14052949	A/G	Synonymous	0.18	F	315	Alt. allele only found in southern Africa (AAF = 0.22–0.52)
X:14061092	C/T	Intronic SRV	0.02	-	-	Alt. allele only present in The Gambia (AAF = 0.14)
X:14063390	A/G	Synonymous	0.02	L	266	Alt. allele only found in Ethiopia (AAF = 0.22)
X:14064963	C/T	Synonymous; SRV	0.02	E	232	Alt. allele only present in The Gambia (AAF = 0.14)
X:14065002	G/A	Synonymous	0.01	R	219	Alt. allele only present in East Africa (AAF = 0.13–0.25)
**X:10465076**	**C/CT CT/C**	**Splice Acceptor**	**0.08 0.34**	**-**	**-**	**Only present in the VRC, expected to be deleterious.**
X:14067819	T/C	Intronic SRV	0.04	-	-	Alt. allele only found in southern Africa (AAF = 0.03–0.13)
X:14077504	A/G	Synonymous	0.02	L	45	Alt. allele only present in Ethiopia (AAF = 0.25)
X:14077524*	A/G	Synonymous	0.61	L	39	Fixation of reference allele in *Ch*. *sabaeus* (AAF_VRC_ = 0.01)
X:14077531	G/A	Synonymous	0.01	A	36	Alt. allele only present in Zambia (AAF = 0.06)
X:14077550*	**T/C**	**Missense**	**0.13**	**D/G**	**30**	**Alt. allele only found in *Ch*. *sabaeus* (AAF = 0.01–1.00; AAF**_**VRC**_ **= 0.36)**

Emboldened, shaded text indicates coding regions or residues critical to SARS-CoV-2 binding. AAF = alternative allele frequency for the full sample. AA = change in amino acid residue predicted to accompany sequence variation. Pos_AA_ = amino acid position in the protein. SRV = splice region variant. Asterisks (*) = variant also present in the Vervet Research Colony at Wake Forest School of Medicine. AAF_VRC_ = alternative allele frequency in the VRC.

^a^For population-specific values of AAF see S1 Table.

Savanna monkeys in the extended pedigree of Caribbean-origin *Ch*. *sabaeus* in the VRC showed marked variation in *ACE2*. A total of eight *ACE2* variants are segregating in the pedigree: 2 splice acceptor site variants, 4 missense variants, and 2 synonymous variants. The two splice acceptor site variants are located at X:14,065,076 (at the end of a polyT tract) and were not observed in the wild monkeys. Both variants are common in the VRC (AAF_VRC_ = 0.08 and AAF_VRC_ = 0.34), and both are expected to be protein truncating variants with potential deleterious effects on the protein. These novel variants might have emerged in the VRC as *de novo* mutations, or may be present in wild populations at very low frequencies not captured with our moderate sample sizes. That the VRC pedigree is founded from a relatively small number of individuals, themselves originating from the founder population on St. Kitts, followed by the expansion of the pedigree might have led to an increased frequency of such rare variants. All four missense variants observed in wild *Ch*. *sabaeus* (X:14,035,353, X:14,035,374, X:14,045,023, and X:14,077,550 causing Asp30Gly) are also present the VRC.

We also examined the genetic variation in *TMPRSS2* both in the wild populations and VRC (S2 and S3 Tables). A total of 15 missense and 21 synonymous variants were observed in the wild populations. In the VRC, one missense, one splice region and 9 synonymous variants were identified, including several variants shared with the East, Central, and South African populations, and all *Ch*. *sabaeus* variants (all of which are synonymous) observed in the wild populations, except one rare variant found in the Gambian population. An additional missense variant and splice variant were observed only in the VRC. Although there is substantially more variation in *TMPRSS2* compared to *ACE2* among savanna monkeys, all retain the functional residues for the cleavage site (R255) and for the catalytic triad (H296, D345, and S441).

## Discussion

The results of this first investigation of SARS-CoV-2 susceptibility in savanna monkeys in both the wild populations and in the VRC pedigree suggest that there are population differences in *ACE2* sequence variation among savanna monkeys, some of which are estimated to yield changes in amino acid residues or exert deleterious effects on the transcript and protein function. The majority of the variants observed in this study in the wild monkeys are unlikely to dramatically alter susceptibility to disease upon exposure to SARS-CoV-2. The lack of frameshift mutations is in agreement with the paucity of protein-truncating variants in the human ortholog observed in gnomAD population data, apparently reflecting the intolerance of this gene against this type of variation in humans [51]. One of these changes (X:14077550; Asp30Gly) occurs at a previously identified critical binding site, part of the SARS-CoV-2 spike receptor binding domain [14]. A similar variant at this site (Asp30Glu, E30) is seen in the *Microcebus murinus* reference genome, and is predicted to confer reduced binding affinity of SARS-CoV-2 to ACE2 receptors (ΔΔG_bind_ = 0.692 kcal/mol) [14]. The Asp30Gly mutation, found in West African savanna monkeys and their Caribbean descendant populations (more commonly called African green monkeys, *Ch*. *sabaeus*), is predicted to destabilize the bond between the virus and the receptor to a greater degree (ΔΔG_bind_ = 0.958 kcal/mol). This suggests that a sizable proportion of the populations of *Ch*. *sabaeus* in West Africa and the Caribbean, those possessing the G30 residue (see S1 Table for population-specific details on prevalence), may have reduced susceptibility to SARS-CoV-2 infection and developing COVID-19-like illness.

Given the lack of clear impact on known functional regions in *TMPRSS2*, it remains to be seen what effect, if any, the population-specific missense variants observed might have on SARS-CoV-2 susceptibility. That expression of *TMPRSS2* is dependent on androgen (AR) expression [24], however, may explain why human males appear to show significantly higher mortality with COVID-19 [52]. We might expect to see a similar, sex-specific pattern of severity in non-human primates. That male savanna monkeys are often bolder than females [53] suggests that they may both be more likely to interact with humans and more vulnerable to severe outcomes from SARS-CoV-2 infection.

Overall, our analysis suggests that savanna monkeys are likely susceptible to SARS-CoV-2 infection, though some populations–notably, those African green monkeys (*Ch*. *sabaeus*) with the Asp30Gly variant–may possess additional resistance to the disease, and that bi-directional infections between humans and savanna monkeys are possible. This high probability for susceptibility suggests that, as in other infected non-human primates (e.g., rhesus macaques) [16] [17] [18], savanna monkeys will likely become ill upon contracting the disease, and, like macaques, may suffer similar symptoms while being infectious to humans. Although this means there is high potential for savanna monkeys to experience concurrent outbreaks of COVID-19-like illness with sympatric human populations, they are unlikely to serve as a long-term reservoir of the disease, and may be less susceptible in the case of re-infection [32]. Additionally, strict quarantines in response to epidemics in nations with high contact between savanna monkeys and humans, like those implemented in March and April of 2020 in South Africa [54], will likely prevent such bi-directional transmissions from occurring as they will also isolate humans from non-human primate populations. Although personal protective equipment is the norm in captive primate facilities and many rescue centers and sanctuaries, similar isolation measures should be considered among those populations when human infection is likely.

We acknowledge that identifying susceptibility or resistance to infection based on receptor usage has limitations, especially with regards to describing severity of disease which has multiple contributing factors, for example the role of inflammatory cytokine responses in immunopathogenesis [55]. However, receptor usage provides an indication of species that could potentially be susceptible [11] [14]. The close proximity of humans and wild non-human primates provides potential for cross-species transmission of pathogens, and for some endangered species this could have devastating effects [14]. Similarly, identifying if non-human primates have the potential to act as intermediates hosts, as for Ebola virus [56], would be important for understanding zoonotic transmission and use of animal models.

Clear steps that must be taken to better understand this potential threat include characterizing the clinical signs of SARS-CoV-2 infection in savanna monkeys, and assessing whether they can develop a COVID-19-like disease state as seen in rhesus macaques. Established captive research colonies, such as those at the Vervet Research Colony of the Wake Forest School of Medicine in the United States (*Ch*. *sabaeus*), the St. Kitts Biomedical Research Foundation in St. Kitts & Nevis (*Ch*. *sabaeus*), and the Medical Research Council in South Africa (*Ch*. *pygerythrus pygerythrus*), would be ideal for such assessments [25] [44]. These colonies may also be critical to the establishment of vaccines and therapies for COVID-19 should the green monkeys serve as a better model of SARS-CoV-2 infection and symptoms than macaque models, as is the case with SARS-CoV [32].

To facilitate such assessments, we characterized *ACE2* and *TMPRSS2* variation in the Vervet Research Colony. All four missense variants present in the wild *Ch*. *sabaeus* populations, including the potential modifying variant Asp30Gly (AAF_VRC_ = 0.36) are segregating in the VRC. The monkeys in the VRC may be useful models for better understanding variable susceptibility to infection with SARS-CoV-2 and developing appropriate interventions in these nonhuman primate populations. Human data collected so far lacks common natural mutations conferring differing efficiencies in coronavirus S-protein binding [57]. From this perspective, a model species with natural genetic variation with potential modulatory effects on the ACE2 role in the SARS-CoV-2 infection is of particular interest. In the VRC, we observed two splice acceptor variants potentially resulting in the truncation of the ACE2 protein. While they may provide a potential natural model for studies of the pathomechanism of the SARS-CoV-2 infection, they should be treated with caution as these variants are located in the repetitive region and require further validation through genotyping animals of interest.

Efforts should also be made to sample widely from wild savanna monkeys–with a focus on those in close contact with humans–in order to assess whether the virus has infected wild primate populations, and what characteristics of the local environment are important to human-monkey transmission. Researchers affiliated with the International Vervet Research Consortium already have contacts and protocols in place to potentially conduct such research in St. Kitts & Nevis, South Africa, and The Gambia [25] [44]. Such assessments could best characterize whether anthroponotic/zoonotic transfer of SARS-CoV-2 is a genuine public health and conservation concern, and predict areas where risk is high for such transmission. This work could better inform local policy with respect to human wildlife interactions. These steps will be essential to assess the potential risk of COVID-19 outbreaks mediated by human-monkey contact or conflict with savanna monkeys in Africa and the Caribbean.

Such assessments are critical, and would be the first steps towards a One Health approach to future monitoring of SARS-CoV-2 [58] [59]. Savanna monkeys predominantly live in nations with generally moderate health infrastructure, which have rapidly prepared but are also predicted to be among the hardest-hit by the current pandemic [60] [61]. Previous cycles of zoonotic disease transmission have already been well-established for other fatal outbreaks in such settings, in non-human primates with significantly lower frequencies of human interaction (e.g., wild apes and Ebola [55]). A clear understanding of potential coincident outbreaks and zoonotic cycles, especially in highly human impacted non-human primate populations, may be important to the long-term management of SARS-CoV-2 in both human health and conservation contexts.

## Supporting information

S1 Table Population-specific allele frequencies for potential functional variants in *ACE2* gene region sequence among wild savanna monkeys. Emboldened taxa and populations show the alternative allele at the given locus.
(DOCX)

S2 Table Potential functional variants in *TMPRSS2* gene region sequence among wild savanna monkeys. Emboldened, shaded text indicates coding regions or residues critical to TMPRSS2 function in relation to SARS-CoV-2. AAF = alternative allele frequency for the full sample. AA = change in amino acid residue predicted to accompany sequence variation. Pos_AA_ = amino acid position in the protein. SRV = splice region variant. Asterisks (*) = variant also present in the Vervet Research Colony at Wake Forest School of Medicine. AAF_VRC_ = alternative allele frequency in the VRC. ^a^ For population-specific AAF values, see S3 Table.
(DOCX)

S3 Table Population-specific allele frequencies for potential functional variants in *TMPRSS2* gene region sequence among wild savanna monkeys. Emboldened taxa and populations show the alternative allele at the given locus.
(DOCX)

S1 Fig (TIF)
