# Supplementary material for: ACE2 and TMPRSS2 variation in savanna monkeys (Chlorocebus spp.): Potential risk for zoonotic/anthroponotic transmission of SARS-CoV-2 and a potential model for functional studies
Source: PLoS One. 2020 Jun 23;15(6):e0235106. doi: 10.1371/journal.pone.0235106 (PMC7310727; doi:10.1371/journal.pone.0235106)
Supplement: S1 Table — Emboldened taxa and populations show the alternative allele at the given locus. (DOCX) [file pone.0235106.s001.docx]

**S1 Table: Population-specific allele frequencies for potential functional variants in *ACE2* gene region sequence among wild savanna monkeys.**

| **Position** | **Variant** | **Consequence** | **Taxon** | **Population** | **n** | **AAF** |
| --- | --- | --- | --- | --- | --- | --- |
| X:14035311 | T/C | Missense | ***Ch. aethiops*** | **Ethiopia** | **16** | **0.94** |
|  |  |  | *Ch. cynosuros* | Zambia | 16 | 0.00 |
|  |  |  | *Ch. p. hilgerti* | Tanzania | 2 | 0.00 |
|  |  |  |  | Kenya | 4 | 0.00 |
|  |  |  | *Ch. p. pygerythrus* | Botswana | 2 | 0.00 |
|  |  |  |  | South Africa | 49 | 0.00 |
|  |  |  | *Ch. sabaeus* | Barbados | 5 | 0.00 |
|  |  |  |  | The Gambia | 22 | 0.00 |
|  |  |  |  | Ghana | 2 | 0.00 |
|  |  |  |  | Nevis | 12 | 0.00 |
|  |  |  |  | St. Kitts | 22 | 0.00 |
|  |  |  | *Ch. tantalus* | Cent. Afr. Rep. | 11 | 0.00 |
| X:14035353* | C/T | Missense | *Ch. aethiops* | Ethiopia | 16 | 0.00 |
|  |  |  | *Ch. cynosuros* | Zambia | 16 | 0.00 |
|  |  |  | *Ch. p. hilgerti* | Tanzania | 2 | 0.00 |
|  |  |  |  | Kenya | 4 | 0.00 |
|  |  |  | *Ch. p. pygerythrus* | Botswana | 2 | 0.00 |
|  |  |  |  | South Africa | 49 | 0.00 |
|  |  |  | ***Ch. sabaeus*** | **Barbados** | **5** | **1.00** |
|  |  |  |  | **The Gambia** | **22** | **0.80** |
|  |  |  |  | **Ghana** | **2** | **1.00** |
|  |  |  |  | **Nevis** | **12** | **1.00** |
|  |  |  |  | **St. Kitts** | **22** | **0.75** |
|  |  |  | *Ch. tantalus* | Cent. Afr. Rep. | 11 | 0.00 |
| X:14035354 | G/A | Synonymous | ***Ch. aethiops*** | **Ethiopia** | **16** | **0.94** |
|  |  |  | *Ch. cynosuros* | Zambia | 16 | 0.00 |
|  |  |  | *Ch. p. hilgerti* | Tanzania | 2 | 0.00 |
|  |  |  |  | Kenya | 4 | 0.00 |
|  |  |  | *Ch. p. pygerythrus* | Botswana | 2 | 0.00 |
|  |  |  |  | South Africa | 49 | 0.00 |
|  |  |  | *Ch. sabaeus* | Barbados | 5 | 0.00 |
|  |  |  |  | The Gambia | 22 | 0.00 |
|  |  |  |  | Ghana | 2 | 0.00 |
|  |  |  |  | Nevis | 12 | 0.00 |
|  |  |  |  | St. Kitts | 22 | 0.00 |
|  |  |  | *Ch. tantalus* | Cent. Afr. Rep. | 11 | 0.00 |
| X:14035357 | G/A | Synonymous | ***Ch. aethiops*** | **Ethiopia** | **16** | **0.16** |
|  |  |  | *Ch. cynosuros* | Zambia | 16 | 0.00 |
|  |  |  | *Ch. p. hilgerti* | Tanzania | 2 | 0.00 |
|  |  |  |  | Kenya | 4 | 0.00 |
|  |  |  | *Ch. p. pygerythrus* | Botswana | 2 | 0.00 |
|  |  |  |  | South Africa | 49 | 0.00 |
|  |  |  | *Ch. sabaeus* | Barbados | 5 | 0.00 |
|  |  |  |  | The Gambia | 22 | 0.00 |
|  |  |  |  | Ghana | 2 | 0.00 |
|  |  |  |  | Nevis | 12 | 0.00 |
|  |  |  |  | St. Kitts | 22 | 0.00 |
|  |  |  | *Ch. tantalus* | Cent. Afr. Rep. | 11 | 0.00 |
| X:14035374* | T/C | Missense | ***Ch. aethiops*** | **Ethiopia** | **16** | **1.00** |
|  |  |  | ***Ch. cynosuros*** | **Zambia** | **16** | **1.00** |
|  |  |  | ***Ch. p. hilgerti*** | **Tanzania** | **2** | **1.00** |
|  |  |  |  | **Kenya** | **4** | **1.00** |
|  |  |  | ***Ch. p. pygerythrus*** | **Botswana** | **2** | **1.00** |
|  |  |  |  | **South Africa** | **49** | **1.00** |
|  |  |  | ***Ch. sabaeus*** | **Barbados** | **5** | **1.00** |
|  |  |  |  | **The Gambia** | **22** | **1.00** |
|  |  |  |  | **Ghana** | **2** | **1.00** |
|  |  |  |  | **Nevis** | **12** | **1.00** |
|  |  |  |  | **St. Kitts** | **22** | **0.75** |
|  |  |  | ***Ch. tantalus*** | **Cent. Afr. Rep.** | **11** | **1.00** |
| X:14041898 | T/C | Synonymous | *Ch. aethiops* | Ethiopia | 16 | 0.00 |
|  |  |  | ***Ch. cynosuros*** | **Zambia** | **16** | **0.86** |
|  |  |  | *Ch. p. hilgerti* | Tanzania | 2 | 0.00 |
|  |  |  |  | Kenya | 4 | 0.00 |
|  |  |  | ***Ch. p. pygerythrus*** | **Botswana** | **2** | **1.00** |
|  |  |  |  | **South Africa** | **49** | **0.36** |
|  |  |  | *Ch. sabaeus* | Barbados | 5 | 0.00 |
|  |  |  |  | The Gambia | 22 | 0.00 |
|  |  |  |  | Ghana | 2 | 0.00 |
|  |  |  |  | Nevis | 12 | 0.00 |
|  |  |  |  | St. Kitts | 22 | 0.00 |
|  |  |  | *Ch. tantalus* | Cent. Afr. Rep. | 11 | 0.00 |
| X:14043283 | G/C | Synonymous | *Ch. aethiops* | Ethiopia | 16 | 0.00 |
|  |  |  | *Ch. cynosuros* | Zambia | 16 | 0.00 |
|  |  |  | *Ch. p. hilgerti* | Tanzania | 2 | 0.00 |
|  |  |  |  | Kenya | 4 | 0.00 |
|  |  |  | ***Ch. p. pygerythrus*** | Botswana | 2 | 0.00 |
|  |  |  |  | **South Africa** | **49** | **0.04** |
|  |  |  | *Ch. sabaeus* | Barbados | 5 | 0.00 |
|  |  |  |  | The Gambia | 22 | 0.00 |
|  |  |  |  | Ghana | 2 | 0.00 |
|  |  |  |  | Nevis | 12 | 0.00 |
|  |  |  |  | St. Kitts | 22 | 0.00 |
|  |  |  | ***Ch. tantalus*** | **Cent.Afr.Rep.** | **11** | **0.36** |
| X:14043289* | T/G | Synonymous | *Ch. aethiops* | Ethiopia | 16 | 0.00 |
|  |  |  | ***Ch. cynosuros*** | **Zambia** | **16** | **0.03** |
|  |  |  | *Ch. p. hilgerti* | Tanzania | 2 | 0.00 |
|  |  |  |  | Kenya | 4 | 0.00 |
|  |  |  | ***Ch. p. pygerythrus*** | Botswana | 2 | 0.00 |
|  |  |  |  | **South Africa** | **49** | **0.14** |
|  |  |  | ***Ch. sabaeus*** | Barbados | 5 | 0.00 |
|  |  |  |  | The Gambia | 22 | 0.00 |
|  |  |  |  | **Ghana** | **2** | **0.50** |
|  |  |  |  | Nevis | 12 | 0.00 |
|  |  |  |  | St. Kitts | 22 | 0.00 |
|  |  |  | ***Ch. tantalus*** | **Cent.Afr.Rep.** | **11** | **0.05** |
| X:14043304 | G/A | Synonymous | *Ch. aethiops* | Ethiopia | 16 | 0.00 |
|  |  |  | *Ch. cynosuros* | Zambia | 16 | 0.00 |
|  |  |  | *Ch. p. hilgerti* | Tanzania | 2 | 0.00 |
|  |  |  |  | Kenya | 4 | 0.00 |
|  |  |  | ***Ch. p. pygerythrus*** | Botswana | 2 | 0.00 |
|  |  |  |  | **South Africa** | **49** | **0.10** |
|  |  |  | *Ch. sabaeus* | Barbados | 5 | 0.00 |
|  |  |  |  | The Gambia | 22 | 0.00 |
|  |  |  |  | Ghana | 2 | 0.00 |
|  |  |  |  | Nevis | 12 | 0.00 |
|  |  |  |  | St. Kitts | 22 | 0.00 |
|  |  |  | *Ch. tantalus* | Cent. Afr. Rep. | 11 | 0.00 |
| X:14043773 | T/A | Intronic SRV | ***Ch. aethiops*** | **Ethiopia** | **16** | **1.00** |
|  |  |  | ***Ch. cynosuros*** | **Zambia** | **16** | **0.97** |
|  |  |  | ***Ch. p. hilgerti*** | **Tanzania** | **2** | **1.00** |
|  |  |  |  | **Kenya** | **4** | **1.00** |
|  |  |  | ***Ch. p. pygerythrus*** | **Botswana** | **2** | **1.00** |
|  |  |  |  | **South Africa** | **49** | **0.86** |
|  |  |  | *Ch. sabaeus* | Barbados | 5 | 0.00 |
|  |  |  |  | The Gambia | 22 | 0.00 |
|  |  |  |  | Ghana | 2 | 0.00 |
|  |  |  |  | Nevis | 12 | 0.00 |
|  |  |  |  | St. Kitts | 22 | 0.00 |
|  |  |  | ***Ch. tantalus*** | **Cent.Afr.Rep.** | **11** | **0.73** |
| X:14043797 | T/A | Missense | ***Ch. aethiops*** | **Ethiopia** | **16** | **0.19** |
|  |  |  | *Ch. cynosuros* | Zambia | 16 | 0.00 |
|  |  |  | *Ch. p. hilgerti* | Tanzania | 2 | 0.00 |
|  |  |  |  | Kenya | 4 | 0.00 |
|  |  |  | *Ch. p. pygerythrus* | Botswana | 2 | 0.00 |
|  |  |  |  | South Africa | 49 | 0.00 |
|  |  |  | *Ch. sabaeus* | Barbados | 5 | 0.00 |
|  |  |  |  | The Gambia | 22 | 0.00 |
|  |  |  |  | Ghana | 2 | 0.00 |
|  |  |  |  | Nevis | 12 | 0.00 |
|  |  |  |  | St. Kitts | 22 | 0.00 |
|  |  |  | *Ch. tantalus* | Cent. Afr. Rep. | 11 | 0.00 |
| X:14045023* | C/G | Missense | ***Ch. aethiops*** | **Ethiopia** | **16** | **1.00** |
|  |  |  | ***Ch. cynosuros*** | **Zambia** | **16** | **1.00** |
|  |  |  | ***Ch. p. hilgerti*** | **Tanzania** | **2** | **1.00** |
|  |  |  |  | **Kenya** | **4** | **1.00** |
|  |  |  | ***Ch. p. pygerythrus*** | **Botswana** | **2** | **1.00** |
|  |  |  |  | **South Africa** | **49** | **1.00** |
|  |  |  | ***Ch. sabaeus*** | Barbados | 5 | 0.00 |
|  |  |  |  | The Gambia | 22 | 0.00 |
|  |  |  |  | **Ghana** | **2** | **0.50** |
|  |  |  |  | Nevis | 12 | 0.00 |
|  |  |  |  | St. Kitts | 22 | 0.00 |
|  |  |  | ***Ch. tantalus*** | **Cent.Afr.Rep.** | **11** | **1.00** |
| X:14049928 | G/A | Synonymous | *Ch. aethiops* | Ethiopia | 16 | 0.00 |
|  |  |  | ***Ch. cynosuros*** | **Zambia** | **16** | **0.03** |
|  |  |  | ***Ch. p. hilgerti*** | Tanzania | 2 | 0.00 |
|  |  |  |  | **Kenya** | **4** | **0.13** |
|  |  |  | *Ch. p. pygerythrus* | Botswana | 2 | 0.00 |
|  |  |  |  | South Africa | 49 | 0.00 |
|  |  |  | *Ch. sabaeus* | Barbados | 5 | 0.00 |
|  |  |  |  | The Gambia | 22 | 0.00 |
|  |  |  |  | Ghana | 2 | 0.00 |
|  |  |  |  | Nevis | 12 | 0.00 |
|  |  |  |  | St. Kitts | 22 | 0.00 |
|  |  |  | *Ch. tantalus* | Cent. Afr. Rep. | 11 | 0.00 |
| X:14052949 | A/G | Synonymous | *Ch. aethiops* | Ethiopia | 16 | 0.00 |
|  |  |  | ***Ch. cynosuros*** | **Zambia** | **16** | **0.22** |
|  |  |  | *Ch. p. hilgerti* | Tanzania | 2 | 0.00 |
|  |  |  |  | Kenya | 4 | 0.00 |
|  |  |  | ***Ch. p. pygerythrus*** | Botswana | 2 | 0.00 |
|  |  |  |  | **South Africa** | **49** | **0.52** |
|  |  |  | *Ch. sabaeus* | Barbados | 5 | 0.00 |
|  |  |  |  | The Gambia | 22 | 0.00 |
|  |  |  |  | Ghana | 2 | 0.00 |
|  |  |  |  | Nevis | 12 | 0.00 |
|  |  |  |  | St. Kitts | 22 | 0.00 |
|  |  |  | *Ch. tantalus* | Cent. Afr. Rep. | 11 | 0.00 |
| X:14061092 | C/T | Intronic SRV | *Ch. aethiops* | Ethiopia | 16 | 0.00 |
|  |  |  | *Ch. cynosuros* | Zambia | 16 | 0.00 |
|  |  |  | *Ch. p. hilgerti* | Tanzania | 2 | 0.00 |
|  |  |  |  | Kenya | 4 | 0.00 |
|  |  |  | *Ch. p. pygerythrus* | Botswana | 2 | 0.00 |
|  |  |  |  | South Africa | 49 | 0.00 |
|  |  |  | *Ch. sabaeus* | Barbados | 5 | 0.00 |
|  |  |  |  | The Gambia | 22 | 0.14 |
|  |  |  |  | Ghana | 2 | 0.00 |
|  |  |  |  | Nevis | 12 | 0.00 |
|  |  |  |  | St. Kitts | 22 | 0.00 |
|  |  |  | *Ch. tantalus* | Cent. Afr. Rep. | 11 | 0.00 |
| X:14063390 | A/G | Synonymous | ***Ch. aethiops*** | **Ethiopia** | **16** | **0.22** |
|  |  |  | *Ch. cynosuros* | Zambia | 16 | 0.00 |
|  |  |  | *Ch. p. hilgerti* | Tanzania | 2 | 0.00 |
|  |  |  |  | Kenya | 4 | 0.00 |
|  |  |  | *Ch. p. pygerythrus* | Botswana | 2 | 0.00 |
|  |  |  |  | South Africa | 49 | 0.00 |
|  |  |  | *Ch. sabaeus* | Barbados | 5 | 0.00 |
|  |  |  |  | The Gambia | 22 | 0.00 |
|  |  |  |  | Ghana | 2 | 0.00 |
|  |  |  |  | Nevis | 12 | 0.00 |
|  |  |  |  | St. Kitts | 22 | 0.00 |
|  |  |  | *Ch. tantalus* | Cent. Afr. Rep. | 11 | 0.00 |
| X:14064963 | C/T | Synonymous; SRV | *Ch. aethiops* | Ethiopia | 16 | 0.00 |
|  |  |  | *Ch. cynosuros* | Zambia | 16 | 0.00 |
|  |  |  | *Ch. p. hilgerti* | Tanzania | 2 | 0.00 |
|  |  |  |  | Kenya | 4 | 0.00 |
|  |  |  | *Ch. p. pygerythrus* | Botswana | 2 | 0.00 |
|  |  |  |  | South Africa | 49 | 0.00 |
|  |  |  | ***Ch. sabaeus*** | Barbados | 5 | 0.00 |
|  |  |  |  | **The Gambia** | **22** | **0.14** |
|  |  |  |  | Ghana | 2 | 0.00 |
|  |  |  |  | Nevis | 12 | 0.00 |
|  |  |  |  | St. Kitts | 22 | 0.00 |
|  |  |  | *Ch. tantalus* | Cent. Afr. Rep. | 11 | 0.00 |
| X:14065002 | G/A | Synonymous | *Ch. aethiops* | Ethiopia | 16 | 0.00 |
|  |  |  | *Ch. cynosuros* | Zambia | 16 | 0.00 |
|  |  |  | ***Ch. p. hilgerti*** | **Tanzania** | **2** | **0.25** |
|  |  |  |  | **Kenya** | **4** | **0.13** |
|  |  |  | *Ch. p. pygerythrus* | Botswana | 2 | 0.00 |
|  |  |  |  | South Africa | 49 | 0.00 |
|  |  |  | *Ch. sabaeus* | Barbados | 5 | 0.00 |
|  |  |  |  | The Gambia | 22 | 0.00 |
|  |  |  |  | Ghana | 2 | 0.00 |
|  |  |  |  | Nevis | 12 | 0.00 |
|  |  |  |  | St. Kitts | 22 | 0.00 |
|  |  |  | *Ch. tantalus* | Cent. Afr. Rep. | 11 | 0.00 |
| X:14067819 | T/C | Intronic SRV | *Ch. aethiops* | Ethiopia | 16 | 0.00 |
|  |  |  | ***Ch. cynosuros*** | **Zambia** | **16** | **0.03** |
|  |  |  | *Ch. p. hilgerti* | Tanzania | 2 | 0.00 |
|  |  |  |  | Kenya | 4 | 0.00 |
|  |  |  | ***Ch. p. pygerythrus*** | Botswana | 2 | 0.00 |
|  |  |  |  | **South Africa** | **49** | **0.13** |
|  |  |  | *Ch. sabaeus* | Barbados | 5 | 0.00 |
|  |  |  |  | The Gambia | 22 | 0.00 |
|  |  |  |  | Ghana | 2 | 0.00 |
|  |  |  |  | Nevis | 12 | 0.00 |
|  |  |  |  | St. Kitts | 22 | 0.00 |
|  |  |  | *Ch. tantalus* | Cent. Afr. Rep. | 11 | 0.00 |
| X:14077504 | A/G | Synonymous | ***Ch. aethiops*** | **Ethiopia** | **16** | **0.25** |
|  |  |  | *Ch. cynosuros* | Zambia | 16 | 0.00 |
|  |  |  | *Ch. p. hilgerti* | Tanzania | 2 | 0.00 |
|  |  |  |  | Kenya | 4 | 0.00 |
|  |  |  | *Ch. p. pygerythrus* | Botswana | 2 | 0.00 |
|  |  |  |  | South Africa | 49 | 0.00 |
|  |  |  | *Ch. sabaeus* | Barbados | 5 | 0.00 |
|  |  |  |  | The Gambia | 22 | 0.00 |
|  |  |  |  | Ghana | 2 | 0.00 |
|  |  |  |  | Nevis | 12 | 0.00 |
|  |  |  |  | St. Kitts | 22 | 0.00 |
|  |  |  | *Ch. tantalus* | Cent. Afr. Rep. | 11 | 0.00 |
| X:14077524* | A/G | Synonymous | ***Ch. aethiops*** | **Ethiopia** | **16** | **1.00** |
|  |  |  | ***Ch. cynosuros*** | **Zambia** | **16** | **1.00** |
|  |  |  | ***Ch. p. hilgerti*** | **Tanzania** | **2** | **1.00** |
|  |  |  |  | **Kenya** | **4** | **1.00** |
|  |  |  | ***Ch. p. pygerythrus*** | **Botswana** | **2** | **1.00** |
|  |  |  |  | **South Africa** | **49** | **1.00** |
|  |  |  | *Ch. sabaeus* | Barbados | 5 | 0.00 |
|  |  |  |  | The Gambia | 22 | 0.00 |
|  |  |  |  | Ghana | 2 | 0.00 |
|  |  |  |  | Nevis | 12 | 0.00 |
|  |  |  |  | St. Kitts | 22 | 0.00 |
|  |  |  | ***Ch. tantalus*** | **Cent.Afr.Rep.** | **11** | **0.96** |
| X:14077531 | G/A | Synonymous | *Ch. aethiops* | Ethiopia | 16 | 0.00 |
|  |  |  | ***Ch. cynosuros*** | **Zambia** | **16** | **0.06** |
|  |  |  | *Ch. p. hilgerti* | Tanzania | 2 | 0.00 |
|  |  |  |  | Kenya | 4 | 0.00 |
|  |  |  | *Ch. p. pygerythrus* | Botswana | 2 | 0.00 |
|  |  |  |  | South Africa | 49 | 0.00 |
|  |  |  | *Ch. sabaeus* | Barbados | 5 | 0.00 |
|  |  |  |  | The Gambia | 22 | 0.00 |
|  |  |  |  | Ghana | 2 | 0.00 |
|  |  |  |  | Nevis | 12 | 0.00 |
|  |  |  |  | St. Kitts | 22 | 0.00 |
|  |  |  | *Ch. tantalus* | Cent. Afr. Rep. | 11 | 0.00 |
| X:14077550* | T/C | Missense | *Ch. aethiops* | Ethiopia | 16 | 0.00 |
|  |  |  | *Ch. cynosuros* | Zambia | 16 | 0.00 |
|  |  |  | *Ch. p. hilgerti* | Tanzania | 2 | 0.00 |
|  |  |  |  | Kenya | 4 | 0.00 |
|  |  |  | *Ch. p. pygerythrus* | Botswana | 2 | 0.00 |
|  |  |  |  | South Africa | 49 | 0.00 |
|  |  |  | ***Ch. sabaeus*** | **Barbados** | **5** | **1.00** |
|  |  |  |  | **The Gambia** | **22** | **0.09** |
|  |  |  |  | Ghana | 2 | 0.00 |
|  |  |  |  | **Nevis** | **12** | **0.29** |
|  |  |  |  | **St. Kitts** | **22** | **0.48** |
|  |  |  | *Ch. tantalus* | Cent. Afr. Rep. | 11 | 0.00 |

Emboldened taxa and populations show the alternative allele at the given locus.
