# Supplementary material for: ACE2 and TMPRSS2 variation in savanna monkeys (Chlorocebus spp.): Potential risk for zoonotic/anthroponotic transmission of SARS-CoV-2 and a potential model for functional studies
Source: PLoS One. 2020 Jun 23;15(6):e0235106. doi: 10.1371/journal.pone.0235106 (PMC7310727; doi:10.1371/journal.pone.0235106)
Supplement: S2 Table — Emboldened, shaded text indicates coding regions or residues critical to TMPRSS2 function in relation to SARS-CoV-2. AAF = alternative allele frequency for the full sample. AA = change in amino acid residue predicted to accompany sequence variation. PosAA = amino acid position in the protein. SRV = splice region variant. Asterisks (*) = variant also present in the Vervet Research Colony at Wake Forest School of Medicine. AAFVRC = alternative allele frequency in the VRC. a For population-specific AAF values, see S3 Table. (DOCX) [file pone.0235106.s002.docx]

**S2 Table: Potential functional variants in *TMPRSS2* gene region sequence among wild savanna monkeys.**

| **Position** | **Variant** | **Consequence** | **AAF^a^** | **AA** | **Pos_AA_** | **Notes** |
| --- | --- | --- | --- | --- | --- | --- |
| 2:85398708 | T/A | Missense | 0.02 | Q/L | 21 | Alt. allele only present in *Ch. tantalus* (AAF=0.27) |
| 2:85398626* | A/G | Synonymous | 0.61 | P | 48 | Ref. allele fixed in *Ch. sabaeus* sample (AAF_VRC_ = 0.01) |
| 2:85398623 | G/A | Synonymous | 0.01 | V | 49 | Alt. allele only present in *Ch. tantalus* (AAF=0.09) |
| 2:85398605* | G/A | Synonymous | 0.05 | S | 55 | Alt. allele only present in *Ch. sabaeus* (AAF=0.02-0.70; AAF_VRC_=0.12) |
| 2:85398580 | C/T | Missense | 0.03 | A/T | 64 | Alt. allele only present in *Ch. p. hilgerti* (AAF=0.50-0.88) |
| 2:85398554* | C/T | Synonymous | 0.01 | P | 72 | Alt. allele only present in *Ch. tantalus* (AAF=0.14; AAF_VRC_=0.01) |
| 2:85393763 | C/A | Missense | 0.01 | A/S | 93 | Alt. allele only present in *Ch. aethiops* (AAF=0.09) |
| 2:85393760 | C/T | Missense | 0.05 | V/I | 94 | Alt. allele only present in South African *Ch. p. pygerythrus* (AAF=0.16) |
| 2:85393737 | G/A | Synonymous | 0.05 | A | 101 | Alt. allele present in Southern Africa (AAF=0.10-0.16) |
| 2:85392475 | T/G | Missense | 0.01 | K/Q | 112 | Alt. allele only present in *Ch. cynosuros* (AAF=0.13) |
| 2:85392399 | G/A | Missense | 0.05 | S/L | 137 | Alt. allele present in Southern Africa (AAF=0.03-0.14) |
| 2:85392389* | T/C | Synonymous | 0.97 | P | 140 | Ref. allele only present in East and Central Africa (RAF=0.03-0.50; AAF_VRC_=0.99) |
| 2:85392377 | G/A | Synonymous | 0.01 | D | 144 | Alt. allele only present in *Ch. tantalus* (AAF=0.18) |
| 2:85387548 | G/A | Missense | 0.01 | H/Y | 169 | Alt. allele only present in *Ch. aethiops* (AAF=0.06) |
| 2:85387535 | C/T | Missense | 0.06 | R/Q | 173 | AAF=0.01-0.41 |
| 2:85387509 | G/T | Synonymous | 0.01 | R | 182 | Alt. allele only present in *Ch. cynosuros* (AAF=0.13) |
| 2:85387504 | C/T | Synonymous | 0.01 | A | 183 | Alt. allele only present in *Ch. p. hilgerti* (AAF=0.25) |
| 2:85386289* | G/T | Missense | 1 | R/S | 193 | Alt. allele fixed in wild sample. AAF_VRC_=0.99 |
| 2:85386191 | C/T | Synonymous | 0.02 | L | 225 | Alt. allele present in Southern Africa (AAF=0.01-0.16) |
| 2:85381738 | G/A | Synonymous | 0.01 | G | 258 | Alt. allele only present in *Ch. aethiops* (AAF=0.13) |
| 2:85381729 | G/A | Synonymous | 0.01 | N | 261 | Alt. allele only present in *Ch. aethiops* (AAF=0.13) |
| 2:85381715 | G/A | Missense | 0.00 | A/V | 266 | Alt. allele only present in South African *Ch. p. pygerythrus* (AAF=0.01) |
| 2:85381669* | G/A | Synonymous | 0.42 | C | 281 | Alt. allele present in all but *Ch. aethiops*  (AAF_VRC_= 0.34) |
| 2:85381639 | G/A | Synonymous | 0.03 | I | 291 | Alt. allele only present in *Ch. aethiops* (AAF=0.13) |
| 2:85378972 | T/A | Missense | 0.03 | M/L | 371 | Alt. allele only present in *Ch. tantalus* (AAF=0.41) |
| 2:85378958* | C/T | Synonymous | 0.00 | P | 375 | Alt. allele only present in *Ch. cynosuros* (MAF=0.03; AAF_VRC_=0.01) |
| 2:85376820 | C/T | Synonymous | 0.00 | L | 397 | Alt. allele only present in Kenya (AAF=0.13) |
| 2:85376790* | C/T | Synonymous | 0.02 | P | 407 | Alt. allele prevalent in St.Kitts (AAF=0.14; AAF_VRC_=0.16) |
| 2:85376730 | G/C | Synonymous | 0.00 | A | 427 | Alt. allele only present in The Gambia (AAF=0.02) |
| 2:85376714 | T/C | Missense | 0.10 | T/A | 433 | Alt. allele only present in *Ch. aethiops* (AAF=0.97) |
| 2:85376712* | G/A | Synonymous | 0.79 | T | 433 | Ref. allele only present in *Ch. aethiops* (AAF=0.03) and *Ch. sabaeus* (AAF=0.50-0.90; AAF_VRC_= 0.62) |
| 2:85376171 | G/A | Synonymous | 0.02 | L | 445 | Alt. allele only present in *Ch. tantalus* (AAF=0.32) |
| 2:85376153 | C/T | Missense | 0.00 | D/N | 451 | Alt. allele only present in *Ch. cynosuros* (AAF=0.03) |
| 2:85376148 | G/C | Synonymous | 0.01 | V | 452 | Alt. allele only present in *Ch. aethiops* (AAF=0.06) |
| 2:85376136 | G/A | Synonymous | 0.02 | I | 456 | Alt. allele only present in *Ch. aethiops* (AAF=0.19) |
| 2:85376069 | C/T | Missense | 0.02 | V/I | 479 | Alt. allele only present in *Ch. cynosuros* (AAF=0.22) |
| 2:85376061* | C/T | Synonymous | 0.10 | T | 481 | Alt. allele prevalent in *Ch. sabaeus* (AAF=0.10-0.50; AAF_VRC_= 0.36) |
| 2:85376038 | C/T | Missense | 0.01 | R/K | 489 | Alt. allele only present in *Ch. cynosuros* (AAF=0.06) |
| 2:85387613* | T/C | Intronic SRV | - | - | - | Alt. allele only present in VRC (AAF_VRC_=0.99) |

Emboldened, shaded text indicates coding regions or residues critical to TMPRSS2 function in relation to SARS-CoV-2. AAF = alternative allele frequency for the full sample. AA = change in amino acid residue predicted to accompany sequence variation. Pos_AA_ = amino acid position in the protein. SRV = splice region variant. Asterisks (*) = variant also present in the Vervet Research Colony at Wake Forest School of Medicine. AAF_VRC_ = alternative allele frequency in the VRC.

^a^ For population-specific AAF values, see Supplementary Table 3.
