# Supplementary material for: ACE2 and TMPRSS2 variation in savanna monkeys (Chlorocebus spp.): Potential risk for zoonotic/anthroponotic transmission of SARS-CoV-2 and a potential model for functional studies
Source: PLoS One. 2020 Jun 23;15(6):e0235106. doi: 10.1371/journal.pone.0235106 (PMC7310727; doi:10.1371/journal.pone.0235106)
Supplement: S3 Table — Emboldened taxa and populations show the alternative allele at the given locus. (DOCX) [file pone.0235106.s003.docx]

**S3 Table: Population-specific allele frequencies for potential functional variants in *TMPRSS2* gene region sequence among wild savanna monkeys.**

| **Position** | **Variant** | **Consequence** | **Taxon** | **Population** | **n** | **AAF** |
| --- | --- | --- | --- | --- | --- | --- |
| 2:85398708 | T/A | Missense | *Ch. aethiops* | Ethiopia | 16 | 0.00 |
|  |  |  | *Ch. cynosuros* | Zambia | 16 | 0.00 |
|  |  |  | *Ch. p. hilgerti* | Tanzania | 2 | 0.00 |
|  |  |  |  | Kenya | 4 | 0.00 |
|  |  |  | *Ch. p. pygerythrus* | Botswana | 2 | 0.00 |
|  |  |  |  | South Africa | 49 | 0.00 |
|  |  |  | *Ch. sabaeus* | Barbados | 5 | 0.00 |
|  |  |  |  | The Gambia | 22 | 0.00 |
|  |  |  |  | Ghana | 2 | 0.00 |
|  |  |  |  | Nevis | 12 | 0.00 |
|  |  |  |  | St. Kitts | 22 | 0.00 |
|  |  |  | ***Ch. tantalus*** | **Cent.Afr.Rep.** | **11** | **0.27** |
| 2:85398626* | A/G | Synonymous | ***Ch. aethiops*** | **Ethiopia** | **16** | **1.00** |
|  |  |  | ***Ch. cynosuros*** | **Zambia** | **16** | **1.00** |
|  |  |  | ***Ch. p. hilgerti*** | **Tanzania** | **2** | **1.00** |
|  |  |  |  | **Kenya** | **4** | **1.00** |
|  |  |  | ***Ch. p. pygerythrus*** | **Botswana** | **2** | **1.00** |
|  |  |  |  | **South Africa** | **49** | **1.00** |
|  |  |  | *Ch. sabaeus* | Barbados | 5 | 0.00 |
|  |  |  |  | The Gambia | 22 | 0.00 |
|  |  |  |  | Ghana | 2 | 0.00 |
|  |  |  |  | Nevis | 12 | 0.00 |
|  |  |  |  | St. Kitts | 22 | 0.00 |
|  |  |  | ***Ch. tantalus*** | **Cent.Afr.Rep.** | **11** | **1.00** |
| 2:85398623 | G/A | Synonymous | *Ch. aethiops* | Ethiopia | 16 | 0.00 |
|  |  |  | *Ch. cynosuros* | Zambia | 16 | 0.00 |
|  |  |  | *Ch. p. hilgerti* | Tanzania | 2 | 0.00 |
|  |  |  |  | Kenya | 4 | 0.00 |
|  |  |  | *Ch. p. pygerythrus* | Botswana | 2 | 0.00 |
|  |  |  |  | South Africa | 49 | 0.00 |
|  |  |  | *Ch. sabaeus* | Barbados | 5 | 0.00 |
|  |  |  |  | The Gambia | 22 | 0.00 |
|  |  |  |  | Ghana | 2 | 0.00 |
|  |  |  |  | Nevis | 12 | 0.00 |
|  |  |  |  | St. Kitts | 22 | 0.00 |
|  |  |  | ***Ch. tantalus*** | **Cent.Afr.Rep.** | **11** | **0.09** |
| 2:85398605* | G/A | Synonymous | *Ch. aethiops* | Ethiopia | 16 | 0.00 |
|  |  |  | *Ch. cynosuros* | Zambia | 16 | 0.00 |
|  |  |  | *Ch. p. hilgerti* | Tanzania | 2 | 0.00 |
|  |  |  |  | Kenya | 4 | 0.00 |
|  |  |  | *Ch. p. pygerythrus* | Botswana | 2 | 0.00 |
|  |  |  |  | South Africa | 49 | 0.00 |
|  |  |  | ***Ch. sabaeus*** | **Barbados** | **5** | **0.70** |
|  |  |  |  | **The Gambia** | **22** | **0.02** |
|  |  |  |  | Ghana | 2 | 0.00 |
|  |  |  |  | **Nevis** | **12** | **0.13** |
|  |  |  |  | **St. Kitts** | **22** | **0.09** |
|  |  |  | *Ch. tantalus* | Cent. Afr. Rep. | 11 | 0.00 |
| 2:85398580 | C/T | Missense | *Ch. aethiops* | Ethiopia | 16 | 0.00 |
|  |  |  | *Ch. cynosuros* | Zambia | 16 | 0.00 |
|  |  |  | ***Ch. p. hilgerti*** | **Tanzania** | **2** | **0.50** |
|  |  |  |  | **Kenya** | **4** | **0.88** |
|  |  |  | *Ch. p. pygerythrus* | Botswana | 2 | 0.00 |
|  |  |  |  | South Africa | 49 | 0.00 |
|  |  |  | *Ch. sabaeus* | Barbados | 5 | 0.00 |
|  |  |  |  | The Gambia | 22 | 0.00 |
|  |  |  |  | Ghana | 2 | 0.00 |
|  |  |  |  | Nevis | 12 | 0.00 |
|  |  |  |  | St. Kitts | 22 | 0.00 |
|  |  |  | *Ch. tantalus* | Cent. Afr. Rep. | 11 | 0.00 |
| 2:85398554* | C/T | Synonymous | *Ch. aethiops* | Ethiopia | 16 | 0.00 |
|  |  |  | *Ch. cynosuros* | Zambia | 16 | 0.00 |
|  |  |  | *Ch. p. hilgerti* | Tanzania | 2 | 0.00 |
|  |  |  |  | Kenya | 4 | 0.00 |
|  |  |  | *Ch. p. pygerythrus* | Botswana | 2 | 0.00 |
|  |  |  |  | South Africa | 49 | 0.00 |
|  |  |  | *Ch. sabaeus* | Barbados | 5 | 0.00 |
|  |  |  |  | The Gambia | 22 | 0.00 |
|  |  |  |  | Ghana | 2 | 0.00 |
|  |  |  |  | Nevis | 12 | 0.00 |
|  |  |  |  | St. Kitts | 22 | 0.00 |
|  |  |  | ***Ch. tantalus*** | **Cent.Afr.Rep.** | **11** | **0.14** |
| 2:85393763 | C/A | Missense | ***Ch. aethiops*** | **Ethiopia** | **16** | **0.09** |
|  |  |  | *Ch. cynosuros* | Zambia | 16 | 0.00 |
|  |  |  | *Ch. p. hilgerti* | Tanzania | 2 | 0.00 |
|  |  |  |  | Kenya | 4 | 0.00 |
|  |  |  | *Ch. p. pygerythrus* | Botswana | 2 | 0.00 |
|  |  |  |  | South Africa | 49 | 0.00 |
|  |  |  | *Ch. sabaeus* | Barbados | 5 | 0.00 |
|  |  |  |  | The Gambia | 22 | 0.00 |
|  |  |  |  | Ghana | 2 | 0.00 |
|  |  |  |  | Nevis | 12 | 0.00 |
|  |  |  |  | St. Kitts | 22 | 0.00 |
|  |  |  | *Ch. tantalus* | Cent.Afr.Rep. | 11 | 0.00 |
| 2:85393760 | C/T | Missense | *Ch. aethiops* | Ethiopia | 16 | 0.00 |
|  |  |  | *Ch. cynosuros* | Zambia | 16 | 0.00 |
|  |  |  | *Ch. p. hilgerti* | Tanzania | 2 | 0.00 |
|  |  |  |  | Kenya | 4 | 0.00 |
|  |  |  | ***Ch. p. pygerythrus*** | Botswana | 2 | 0.00 |
|  |  |  |  | **South Africa** | **49** | **0.16** |
|  |  |  | *Ch. sabaeus* | Barbados | 5 | 0.00 |
|  |  |  |  | The Gambia | 22 | 0.00 |
|  |  |  |  | Ghana | 2 | 0.00 |
|  |  |  |  | Nevis | 12 | 0.00 |
|  |  |  |  | St. Kitts | 22 | 0.00 |
|  |  |  | *Ch. tantalus* | Cent.Afr.Rep. | 11 | 0.00 |
| 2:85393737 | G/A | Synonymous | *Ch. aethiops* | Ethiopia | 16 | 0.00 |
|  |  |  | ***Ch. cynosuros*** | **Zambia** | **16** | **0.16** |
|  |  |  | *Ch. p. hilgerti* | Tanzania | 2 | 0.00 |
|  |  |  |  | Kenya | 4 | 0.00 |
|  |  |  | ***Ch. p. pygerythrus*** | Botswana | 2 | 0.00 |
|  |  |  |  | **South Africa** | **49** | **0.10** |
|  |  |  | *Ch. sabaeus* | Barbados | 5 | 0.00 |
|  |  |  |  | The Gambia | 22 | 0.00 |
|  |  |  |  | Ghana | 2 | 0.00 |
|  |  |  |  | Nevis | 12 | 0.00 |
|  |  |  |  | St. Kitts | 22 | 0.00 |
|  |  |  | *Ch. tantalus* | Cent. Afr. Rep. | 11 | 0.00 |
| 2:85392475 | T/G | Missense | *Ch. aethiops* | Ethiopia | 16 | 0.00 |
|  |  |  | ***Ch. cynosuros*** | **Zambia** | **16** | **0.13** |
|  |  |  | *Ch. p. hilgerti* | Tanzania | 2 | 0.00 |
|  |  |  |  | Kenya | 4 | 0.00 |
|  |  |  | *Ch. p. pygerythrus* | Botswana | 2 | 0.00 |
|  |  |  |  | South Africa | 49 | 0.00 |
|  |  |  | *Ch. sabaeus* | Barbados | 5 | 0.00 |
|  |  |  |  | The Gambia | 22 | 0.00 |
|  |  |  |  | Ghana | 2 | 0.00 |
|  |  |  |  | Nevis | 12 | 0.00 |
|  |  |  |  | St. Kitts | 22 | 0.00 |
|  |  |  | *Ch. tantalus* | Cent. Afr. Rep. | 11 | 0.00 |
| 2:85392399 | G/A | Missense | *Ch. aethiops* | Ethiopia | 16 | 0.00 |
|  |  |  | ***Ch. cynosuros*** | **Zambia** | **16** | **0.03** |
|  |  |  | *Ch. p. hilgerti* | Tanzania | 2 | 0.00 |
|  |  |  |  | Kenya | 4 | 0.00 |
|  |  |  | ***Ch. p. pygerythrus*** | Botswana | 2 | 0.00 |
|  |  |  |  | **South Africa** | **49** | **0.14** |
|  |  |  | *Ch. sabaeus* | Barbados | 5 | 0.00 |
|  |  |  |  | The Gambia | 22 | 0.00 |
|  |  |  |  | Ghana | 2 | 0.00 |
|  |  |  |  | Nevis | 12 | 0.00 |
|  |  |  |  | St. Kitts | 22 | 0.00 |
|  |  |  | *Ch. tantalus* | Cent. Afr. Rep. | 11 | 0.00 |
| 2:85392389* | T/C | Synonymous | ***Ch. aethiops*** | **Ethiopia** | **16** | **0.97** |
|  |  |  | ***Ch. cynosuros*** | **Zambia** | **16** | **1.00** |
|  |  |  | ***Ch. p. hilgerti*** | **Tanzania** | **2** | **0.50** |
|  |  |  |  | **Kenya** | **4** | **0.88** |
|  |  |  | ***Ch. p. pygerythrus*** | **Botswana** | **2** | **1.00** |
|  |  |  |  | **South Africa** | **49** | **1.00** |
|  |  |  | ***Ch. sabaeus*** | **Barbados** | **5** | **1.00** |
|  |  |  |  | **The Gambia** | **22** | **1.00** |
|  |  |  |  | **Ghana** | **2** | **1.00** |
|  |  |  |  | **Nevis** | **12** | **1.00** |
|  |  |  |  | **St. Kitts** | **22** | **1.00** |
|  |  |  | ***Ch. tantalus*** | **Cent.Afr.Rep.** | **11** | **0.73** |
| 2:85392377 | G/A | Synonymous | *Ch. aethiops* | Ethiopia | 16 | 0.00 |
|  |  |  | *Ch. cynosuros* | Zambia | 16 | 0.00 |
|  |  |  | *Ch. p. hilgerti* | Tanzania | 2 | 0.00 |
|  |  |  |  | Kenya | 4 | 0.00 |
|  |  |  | *Ch. p. pygerythrus* | Botswana | 2 | 0.00 |
|  |  |  |  | South Africa | 49 | 0.00 |
|  |  |  | *Ch. sabaeus* | Barbados | 5 | 0.00 |
|  |  |  |  | The Gambia | 22 | 0.00 |
|  |  |  |  | Ghana | 2 | 0.00 |
|  |  |  |  | Nevis | 12 | 0.00 |
|  |  |  |  | St. Kitts | 22 | 0.00 |
|  |  |  | ***Ch. tantalus*** | **Cent.Afr.Rep.** | **11** | **0.18** |
| 2:85387548 | G/A | Missense | ***Ch. aethiops*** | **Ethiopia** | **16** | **0.06** |
|  |  |  | *Ch. cynosuros* | Zambia | 16 | 0.00 |
|  |  |  | *Ch. p. hilgerti* | Tanzania | 2 | 0.00 |
|  |  |  |  | Kenya | 4 | 0.00 |
|  |  |  | *Ch. p. pygerythrus* | Botswana | 2 | 0.00 |
|  |  |  |  | South Africa | 49 | 0.00 |
|  |  |  | *Ch. sabaeus* | Barbados | 5 | 0.00 |
|  |  |  |  | The Gambia | 22 | 0.00 |
|  |  |  |  | Ghana | 2 | 0.00 |
|  |  |  |  | Nevis | 12 | 0.00 |
|  |  |  |  | St. Kitts | 22 | 0.00 |
|  |  |  | *Ch. tantalus* | Cent. Afr. Rep. | 11 | 0.00 |
| 2:85387535 | C/T | Missense | ***Ch. aethiops*** | **Ethiopia** | **16** | **0.41** |
|  |  |  | *Ch. cynosuros* | Zambia | 16 | 0.00 |
|  |  |  | *Ch. p. hilgerti* | Tanzania | 2 | 0.00 |
|  |  |  |  | Kenya | 4 | 0.00 |
|  |  |  | ***Ch. p. pygerythrus*** | Botswana | 2 | 0.00 |
|  |  |  |  | **South Africa** | **49** | **0.01** |
|  |  |  | *Ch. sabaeus* | Barbados | 5 | 0.00 |
|  |  |  |  | The Gambia | 22 | 0.00 |
|  |  |  |  | Ghana | 2 | 0.00 |
|  |  |  |  | Nevis | 12 | 0.00 |
|  |  |  |  | St. Kitts | 22 | 0.00 |
|  |  |  | ***Ch. tantalus*** | **Cent.Afr.Rep.** | **11** | **0.23** |
| 2:85387509 | G/T | Synonymous | *Ch. aethiops* | Ethiopia | 16 | 0.00 |
|  |  |  | ***Ch. cynosuros*** | **Zambia** | **16** | **0.13** |
|  |  |  | *Ch. p. hilgerti* | Tanzania | 2 | 0.00 |
|  |  |  |  | Kenya | 4 | 0.00 |
|  |  |  | *Ch. p. pygerythrus* | Botswana | 2 | 0.00 |
|  |  |  |  | South Africa | 49 | 0.00 |
|  |  |  | *Ch. sabaeus* | Barbados | 5 | 0.00 |
|  |  |  |  | The Gambia | 22 | 0.00 |
|  |  |  |  | Ghana | 2 | 0.00 |
|  |  |  |  | Nevis | 12 | 0.00 |
|  |  |  |  | St. Kitts | 22 | 0.00 |
|  |  |  | *Ch. tantalus* | Cent. Afr. Rep. | 11 | 0.00 |
| 2:85387504 | C/T | Synonymous | *Ch. aethiops* | Ethiopia | 16 | 0.00 |
|  |  |  | *Ch. cynosuros* | Zambia | 16 | 0.00 |
|  |  |  | ***Ch. p. hilgerti*** | **Tanzania** | **2** | **0.25** |
|  |  |  |  | **Kenya** | **4** | **0.25** |
|  |  |  | *Ch. p. pygerythrus* | Botswana | 2 | 0.00 |
|  |  |  |  | South Africa | 49 | 0.00 |
|  |  |  | *Ch. sabaeus* | Barbados | 5 | 0.00 |
|  |  |  |  | The Gambia | 22 | 0.00 |
|  |  |  |  | Ghana | 2 | 0.00 |
|  |  |  |  | Nevis | 12 | 0.00 |
|  |  |  |  | St. Kitts | 22 | 0.00 |
|  |  |  | *Ch. tantalus* | Cent. Afr. Rep. | 11 | 0.00 |
| 2:85386289* | G/T | Missense | *Ch. aethiops* | Ethiopia | 16 | 0.00 |
|  |  |  | *Ch. cynosuros* | Zambia | 16 | 0.00 |
|  |  |  | *Ch. p. hilgerti* | Tanzania | 2 | 0.00 |
|  |  |  |  | Kenya | 4 | 0.00 |
|  |  |  | *Ch. p. pygerythrus* | Botswana | 2 | 0.00 |
|  |  |  |  | South Africa | 49 | 0.00 |
|  |  |  | *Ch. sabaeus* | Barbados | 5 | 0.00 |
|  |  |  |  | The Gambia | 22 | 0.00 |
|  |  |  |  | Ghana | 2 | 0.00 |
|  |  |  |  | Nevis | 12 | 0.00 |
|  |  |  |  | St. Kitts | 22 | 0.00 |
|  |  |  | *Ch. tantalus* | Cent. Afr. Rep. | 11 | 0.00 |
| 2:85386191 | C/T | Synonymous | *Ch. aethiops* | Ethiopia | 16 | 0.00 |
|  |  |  | ***Ch. cynosuros*** | **Zambia** | **16** | **0.16** |
|  |  |  | *Ch. p. hilgerti* | Tanzania | 2 | 0.00 |
|  |  |  |  | Kenya | 4 | 0.00 |
|  |  |  | ***Ch. p. pygerythrus*** | Botswana | 2 | 0.00 |
|  |  |  |  | **South Africa** | **49** | **0.01** |
|  |  |  | *Ch. sabaeus* | Barbados | 5 | 0.00 |
|  |  |  |  | The Gambia | 22 | 0.00 |
|  |  |  |  | Ghana | 2 | 0.00 |
|  |  |  |  | Nevis | 12 | 0.00 |
|  |  |  |  | St. Kitts | 22 | 0.00 |
|  |  |  | *Ch. tantalus* | Cent. Afr. Rep. | 11 | 0.00 |
| 2:85381738 | G/A | Synonymous | ***Ch. aethiops*** | **Ethiopia** | **16** | **0.13** |
|  |  |  | *Ch. cynosuros* | Zambia | 16 | 0.00 |
|  |  |  | *Ch. p. hilgerti* | Tanzania | 2 | 0.00 |
|  |  |  |  | Kenya | 4 | 0.00 |
|  |  |  | *Ch. p. pygerythrus* | Botswana | 2 | 0.00 |
|  |  |  |  | South Africa | 49 | 0.00 |
|  |  |  | *Ch. sabaeus* | Barbados | 5 | 0.00 |
|  |  |  |  | The Gambia | 22 | 0.00 |
|  |  |  |  | Ghana | 2 | 0.00 |
|  |  |  |  | Nevis | 12 | 0.00 |
|  |  |  |  | St. Kitts | 22 | 0.00 |
|  |  |  | *Ch. tantalus* | Cent. Afr. Rep. | 11 | 0.00 |
| 2:85381729 | G/A | Synonymous | ***Ch. aethiops*** | **Ethiopia** | **16** | **0.13** |
|  |  |  | *Ch. cynosuros* | Zambia | 16 | 0.00 |
|  |  |  | *Ch. p. hilgerti* | Tanzania | 2 | 0.00 |
|  |  |  |  | Kenya | 4 | 0.00 |
|  |  |  | *Ch. p. pygerythrus* | Botswana | 2 | 0.00 |
|  |  |  |  | South Africa | 49 | 0.00 |
|  |  |  | *Ch. sabaeus* | Barbados | 5 | 0.00 |
|  |  |  |  | The Gambia | 22 | 0.00 |
|  |  |  |  | Ghana | 2 | 0.00 |
|  |  |  |  | Nevis | 12 | 0.00 |
|  |  |  |  | St. Kitts | 22 | 0.00 |
|  |  |  | *Ch. tantalus* | Cent.Afr.Rep. | 11 | 0.00 |
| 2:85381715 | G/A | Missense | *Ch. aethiops* | Ethiopia | 16 | 0.00 |
|  |  |  | *Ch. cynosuros* | Zambia | 16 | 0.00 |
|  |  |  | *Ch. p. hilgerti* | Tanzania | 2 | 0.00 |
|  |  |  |  | Kenya | 4 | 0.00 |
|  |  |  | ***Ch. p. pygerythrus*** | Botswana | 2 | 0.00 |
|  |  |  |  | **South Africa** | **49** | **0.01** |
|  |  |  | *Ch. sabaeus* | Barbados | 5 | 0.00 |
|  |  |  |  | The Gambia | 22 | 0.00 |
|  |  |  |  | Ghana | 2 | 0.00 |
|  |  |  |  | Nevis | 12 | 0.00 |
|  |  |  |  | St. Kitts | 22 | 0.00 |
|  |  |  | *Ch. tantalus* | Cent. Afr. Rep. | 11 | 0.00 |
| 2:85381669* | G/A | Synonymous | *Ch. aethiops* | Ethiopia | 16 | 0.00 |
|  |  |  | ***Ch. cynosuros*** | **Zambia** | **16** | **0.50** |
|  |  |  | ***Ch. p. hilgerti*** | **Tanzania** | **2** | **0.75** |
|  |  |  |  | **Kenya** | **4** | **0.38** |
|  |  |  | ***Ch. p. pygerythrus*** | **Botswana** | **2** | **0.25** |
|  |  |  |  | **South Africa** | **49** | **0.41** |
|  |  |  | ***Ch. sabaeus*** | **Barbados** | **5** | **0.60** |
|  |  |  |  | **The Gambia** | **22** | **0.57** |
|  |  |  |  | **Ghana** | **2** | **0.75** |
|  |  |  |  | **Nevis** | **12** | **0.54** |
|  |  |  |  | **St. Kitts** | **22** | **0.57** |
|  |  |  | ***Ch. tantalus*** | **Cent. Afr. Rep.** | **11** | **0.14** |
| 2:85381639 | G/A | Synonymous | ***Ch. aethiops*** | **Ethiopia** | **16** | **0.34** |
|  |  |  | *Ch. cynosuros* | Zambia | 16 | 0.00 |
|  |  |  | *Ch. p. hilgerti* | Tanzania | 2 | 0.00 |
|  |  |  |  | Kenya | 4 | 0.00 |
|  |  |  | *Ch. p. pygerythrus* | Botswana | 2 | 0.00 |
|  |  |  |  | South Africa | 49 | 0.00 |
|  |  |  | *Ch. sabaeus* | Barbados | 5 | 0.00 |
|  |  |  |  | The Gambia | 22 | 0.00 |
|  |  |  |  | Ghana | 2 | 0.00 |
|  |  |  |  | Nevis | 12 | 0.00 |
|  |  |  |  | St. Kitts | 22 | 0.00 |
|  |  |  | *Ch. tantalus* | Cent. Afr. Rep. | 11 | 0.00 |
| 2:85378972 | T/A | Missense | *Ch. aethiops* | Ethiopia | 16 | 0.00 |
|  |  |  | *Ch. cynosuros* | Zambia | 16 | 0.00 |
|  |  |  | *Ch. p. hilgerti* | Tanzania | 2 | 0.00 |
|  |  |  |  | Kenya | 4 | 0.00 |
|  |  |  | *Ch. p. pygerythrus* | Botswana | 2 | 0.00 |
|  |  |  |  | South Africa | 49 | 0.00 |
|  |  |  | *Ch. sabaeus* | Barbados | 5 | 0.00 |
|  |  |  |  | The Gambia | 22 | 0.00 |
|  |  |  |  | Ghana | 2 | 0.00 |
|  |  |  |  | Nevis | 12 | 0.00 |
|  |  |  |  | St. Kitts | 22 | 0.00 |
|  |  |  | ***Ch. tantalus*** | **Cent.Afr.Rep.** | **11** | **0.41** |
| 2:85378958* | C/T | Synonymous | *Ch. aethiops* | Ethiopia | 16 | 0.00 |
|  |  |  | ***Ch. cynosuros*** | **Zambia** | **16** | **0.03** |
|  |  |  | *Ch. p. hilgerti* | Tanzania | 2 | 0.00 |
|  |  |  |  | Kenya | 4 | 0.00 |
|  |  |  | *Ch. p. pygerythrus* | Botswana | 2 | 0.00 |
|  |  |  |  | South Africa | 49 | 0.00 |
|  |  |  | *Ch. sabaeus* | Barbados | 5 | 0.00 |
|  |  |  |  | The Gambia | 22 | 0.00 |
|  |  |  |  | Ghana | 2 | 0.00 |
|  |  |  |  | Nevis | 12 | 0.00 |
|  |  |  |  | St. Kitts | 22 | 0.00 |
|  |  |  | *Ch. tantalus* | Cent. Afr. Rep. | 11 | 0.00 |
| 2:85376820 | C/T | Synonymous | *Ch. aethiops* | Ethiopia | 16 | 0.00 |
|  |  |  | *Ch. cynosuros* | Zambia | 16 | 0.00 |
|  |  |  | *Ch. p. hilgerti* | Tanzania | 2 | 0.00 |
|  |  |  |  | Kenya | 4 | 0.13 |
|  |  |  | *Ch. p. pygerythrus* | Botswana | 2 | 0.00 |
|  |  |  |  | South Africa | 49 | 0.00 |
|  |  |  | *Ch. sabaeus* | Barbados | 5 | 0.00 |
|  |  |  |  | The Gambia | 22 | 0.00 |
|  |  |  |  | Ghana | 2 | 0.00 |
|  |  |  |  | Nevis | 12 | 0.00 |
|  |  |  |  | St. Kitts | 22 | 0.00 |
|  |  |  | *Ch. tantalus* | Cent. Afr. Rep. | 11 | 0.00 |
| 2:85376790* | C/T | Synonymous | *Ch. aethiops* | Ethiopia | 16 | 0.00 |
|  |  |  | *Ch. cynosuros* | Zambia | 16 | 0.00 |
|  |  |  | *Ch. p. hilgerti* | Tanzania | 2 | 0.00 |
|  |  |  |  | Kenya | 4 | 0.00 |
|  |  |  | ***Ch. p. pygerythrus*** | Botswana | 2 | 0.00 |
|  |  |  |  | **South Africa** | **49** | **0.01** |
|  |  |  | ***Ch. sabaeus*** | Barbados | 5 | 0.00 |
|  |  |  |  | The Gambia | 22 | 0.00 |
|  |  |  |  | Ghana | 2 | 0.00 |
|  |  |  |  | Nevis | 12 | 0.00 |
|  |  |  |  | **St. Kitts** | **22** | **0.14** |
|  |  |  | *Ch. tantalus* | Cent. Afr. Rep. | 11 | 0.00 |
| 2:85376730 | G/C | Synonymous | *Ch. aethiops* | Ethiopia | 16 | 0.00 |
|  |  |  | *Ch. cynosuros* | Zambia | 16 | 0.00 |
|  |  |  | *Ch. p. hilgerti* | Tanzania | 2 | 0.00 |
|  |  |  |  | Kenya | 4 | 0.00 |
|  |  |  | *Ch. p. pygerythrus* | Botswana | 2 | 0.00 |
|  |  |  |  | South Africa | 49 | 0.00 |
|  |  |  | ***Ch. sabaeus*** | Barbados | 5 | 0.00 |
|  |  |  |  | **The Gambia** | **22** | **0.02** |
|  |  |  |  | Ghana | 2 | 0.00 |
|  |  |  |  | Nevis | 12 | 0.00 |
|  |  |  |  | St. Kitts | 22 | 0.00 |
|  |  |  | *Ch. tantalus* | Cent. Afr. Rep. | 11 | 0.00 |
| 2:85376714 | T/C | Missense | ***Ch. aethiops*** | **Ethiopia** | **16** | **0.97** |
|  |  |  | *Ch. cynosuros* | Zambia | 16 | 0.00 |
|  |  |  | *Ch. p. hilgerti* | Tanzania | 2 | 0.00 |
|  |  |  |  | Kenya | 4 | 0.00 |
|  |  |  | *Ch. p. pygerythrus* | Botswana | 2 | 0.00 |
|  |  |  |  | South Africa | 49 | 0.00 |
|  |  |  | *Ch. sabaeus* | Barbados | 5 | 0.00 |
|  |  |  |  | The Gambia | 22 | 0.00 |
|  |  |  |  | Ghana | 2 | 0.00 |
|  |  |  |  | Nevis | 12 | 0.00 |
|  |  |  |  | St. Kitts | 22 | 0.00 |
|  |  |  | *Ch. tantalus* | Cent. Afr. Rep. | 11 | 0.00 |
| 2:85376712* | G/A | Synonymous | ***Ch. aethiops*** | **Ethiopia** | **16** | **0.03** |
|  |  |  | ***Ch. cynosuros*** | **Zambia** | **16** | **1.00** |
|  |  |  | ***Ch. p. hilgerti*** | **Tanzania** | **2** | **1.00** |
|  |  |  |  | **Kenya** | **4** | **1.00** |
|  |  |  | ***Ch. p. pygerythrus*** | **Botswana** | **2** | **1.00** |
|  |  |  |  | **South Africa** | **49** | **1.00** |
|  |  |  | ***Ch. sabaeus*** | **Barbados** | **5** | **0.90** |
|  |  |  |  | **The Gambia** | **22** | **0.60** |
|  |  |  |  | **Ghana** | **2** | **0.50** |
|  |  |  |  | **Nevis** | **12** | **0.67** |
|  |  |  |  | **St. Kitts** | **22** | **0.77** |
|  |  |  | ***Ch. tantalus*** | **Cent. Afr. Rep.** | **11** | **1.00** |
| 2:85376171 | G/A | Synonymous | *Ch. aethiops* | Ethiopia | 16 | 0.00 |
|  |  |  | *Ch. cynosuros* | Zambia | 16 | 0.00 |
|  |  |  | *Ch. p. hilgerti* | Tanzania | 2 | 0.00 |
|  |  |  |  | Kenya | 4 | 0.00 |
|  |  |  | *Ch. p. pygerythrus* | Botswana | 2 | 0.00 |
|  |  |  |  | South Africa | 49 | 0.00 |
|  |  |  | *Ch. sabaeus* | Barbados | 5 | 0.00 |
|  |  |  |  | The Gambia | 22 | 0.00 |
|  |  |  |  | Ghana | 2 | 0.00 |
|  |  |  |  | Nevis | 12 | 0.00 |
|  |  |  |  | St. Kitts | 22 | 0.00 |
|  |  |  | ***Ch. tantalus*** | **Cent.Afr.Rep.** | **11** | **0.32** |
| 2:85376153 | C/T | Missense | *Ch. aethiops* | Ethiopia | 16 | 0.00 |
|  |  |  | ***Ch. cynosuros*** | **Zambia** | **16** | **0.03** |
|  |  |  | *Ch. p. hilgerti* | Tanzania | 2 | 0.00 |
|  |  |  |  | Kenya | 4 | 0.00 |
|  |  |  | *Ch. p. pygerythrus* | Botswana | 2 | 0.00 |
|  |  |  |  | South Africa | 49 | 0.00 |
|  |  |  | *Ch. sabaeus* | Barbados | 5 | 0.00 |
|  |  |  |  | The Gambia | 22 | 0.00 |
|  |  |  |  | Ghana | 2 | 0.00 |
|  |  |  |  | Nevis | 12 | 0.00 |
|  |  |  |  | St. Kitts | 22 | 0.00 |
|  |  |  | *Ch. tantalus* | Cent. Afr. Rep. | 11 | 0.00 |
| 2:85376148 | G/C | Synonymous | ***Ch. aethiops*** | **Ethiopia** | **16** | **0.06** |
|  |  |  | *Ch. cynosuros* | Zambia | 16 | 0.00 |
|  |  |  | *Ch. p. hilgerti* | Tanzania | 2 | 0.00 |
|  |  |  |  | Kenya | 4 | 0.00 |
|  |  |  | *Ch. p. pygerythrus* | Botswana | 2 | 0.00 |
|  |  |  |  | South Africa | 49 | 0.00 |
|  |  |  | *Ch. sabaeus* | Barbados | 5 | 0.00 |
|  |  |  |  | The Gambia | 22 | 0.00 |
|  |  |  |  | Ghana | 2 | 0.00 |
|  |  |  |  | Nevis | 12 | 0.00 |
|  |  |  |  | St. Kitts | 22 | 0.00 |
|  |  |  | *Ch. tantalus* | Cent. Afr. Rep. | 11 | 0.00 |
| 2:85376136 | G/A | Synonymous | ***Ch. aethiops*** | **Ethiopia** | **16** | **0.19** |
|  |  |  | *Ch. cynosuros* | Zambia | 16 | 0.00 |
|  |  |  | *Ch. p. hilgerti* | Tanzania | 2 | 0.00 |
|  |  |  |  | Kenya | 4 | 0.00 |
|  |  |  | *Ch. p. pygerythrus* | Botswana | 2 | 0.00 |
|  |  |  |  | South Africa | 49 | 0.00 |
|  |  |  | *Ch. sabaeus* | Barbados | 5 | 0.00 |
|  |  |  |  | The Gambia | 22 | 0.00 |
|  |  |  |  | Ghana | 2 | 0.00 |
|  |  |  |  | Nevis | 12 | 0.00 |
|  |  |  |  | St. Kitts | 22 | 0.00 |
|  |  |  | *Ch. tantalus* | Cent. Afr. Rep. | 11 | 0.00 |
| 2:85376069 | C/T | Missense | *Ch. aethiops* | Ethiopia | 16 | 0.00 |
|  |  |  | *Ch. cynosuros* | Zambia | 16 | 0.22 |
|  |  |  | *Ch. p. hilgerti* | Tanzania | 2 | 0.00 |
|  |  |  |  | Kenya | 4 | 0.00 |
|  |  |  | *Ch. p. pygerythrus* | Botswana | 2 | 0.00 |
|  |  |  |  | South Africa | 49 | 0.00 |
|  |  |  | *Ch. sabaeus* | Barbados | 5 | 0.00 |
|  |  |  |  | The Gambia | 22 | 0.00 |
|  |  |  |  | Ghana | 2 | 0.00 |
|  |  |  |  | Nevis | 12 | 0.00 |
|  |  |  |  | St. Kitts | 22 | 0.00 |
|  |  |  | *Ch. tantalus* | Cent. Afr. Rep. | 11 | 0.00 |
| 2:85376061* | C/T | Synonymous | *Ch. aethiops* | Ethiopia | 16 | 0.00 |
|  |  |  | *Ch. cynosuros* | Zambia | 16 | 0.00 |
|  |  |  | ***Ch. p. hilgerti*** | Tanzania | 2 | 0.00 |
|  |  |  |  | **Kenya** | **4** | **0.13** |
|  |  |  | *Ch. p. pygerythrus* | Botswana | 2 | 0.00 |
|  |  |  |  | South Africa | 49 | 0.00 |
|  |  |  | ***Ch. sabaeus*** | **Barbados** | **5** | **0.10** |
|  |  |  |  | **The Gambia** | **22** | **0.25** |
|  |  |  |  | **Ghana** | **2** | **0.50** |
|  |  |  |  | **Nevis** | **12** | **0.33** |
|  |  |  |  | **St. Kitts** | **22** | **0.23** |
|  |  |  | *Ch. tantalus* | Cent. Afr. Rep. | 11 | 0.00 |
| 2:85376038 | C/T | Missense | *Ch. aethiops* | Ethiopia | 16 | 0.00 |
|  |  |  | ***Ch. cynosuros*** | **Zambia** | **16** | **0.06** |
|  |  |  | *Ch. p. hilgerti* | Tanzania | 2 | 0.00 |
|  |  |  |  | Kenya | 4 | 0.00 |
|  |  |  | *Ch. p. pygerythrus* | Botswana | 2 | 0.00 |
|  |  |  |  | South Africa | 49 | 0.00 |
|  |  |  | *Ch. sabaeus* | Barbados | 5 | 0.00 |
|  |  |  |  | The Gambia | 22 | 0.00 |
|  |  |  |  | Ghana | 2 | 0.00 |
|  |  |  |  | Nevis | 12 | 0.00 |
|  |  |  |  | St. Kitts | 22 | 0.00 |
|  |  |  | *Ch. tantalus* | Cent. Afr. Rep. | 11 | 0.00 |
| 2:85387613* | T/C | Intronic SRV | ***Ch. aethiops*** | **Ethiopia** | **16** | **1.00** |
|  |  |  | ***Ch. cynosuros*** | **Zambia** | **16** | **0.84** |
|  |  |  | ***Ch. p. hilgerti*** | **Tanzania** | **2** | **1.00** |
|  |  |  |  | **Kenya** | **4** | **1.00** |
|  |  |  | ***Ch. p. pygerythrus*** | **Botswana** | **2** | **1.00** |
|  |  |  |  | **South Africa** | **49** | **0.91** |
|  |  |  | ***Ch. sabaeus*** | **Barbados** | **5** | **1.00** |
|  |  |  |  | **The Gambia** | **22** | **1.00** |
|  |  |  |  | **Ghana** | **2** | **1.00** |
|  |  |  |  | **Nevis** | **12** | **1.00** |
|  |  |  |  | **St. Kitts** | **22** | **1.00** |
|  |  |  | ***Ch. tantalus*** | **Cent.Afr.Rep.** | **11** | **0.59** |

Emboldened taxa and populations show the alternative allele at the given locus.
