# Supplementary figures and images for: ACE2 and TMPRSS2 variation in savanna monkeys (Chlorocebus spp.): Potential risk for zoonotic/anthroponotic transmission of SARS-CoV-2 and a potential model for functional studies
Source: PLoS One. 2020 Jun 23;15(6):e0235106. doi: 10.1371/journal.pone.0235106 (PMC7310727; doi:10.1371/journal.pone.0235106)

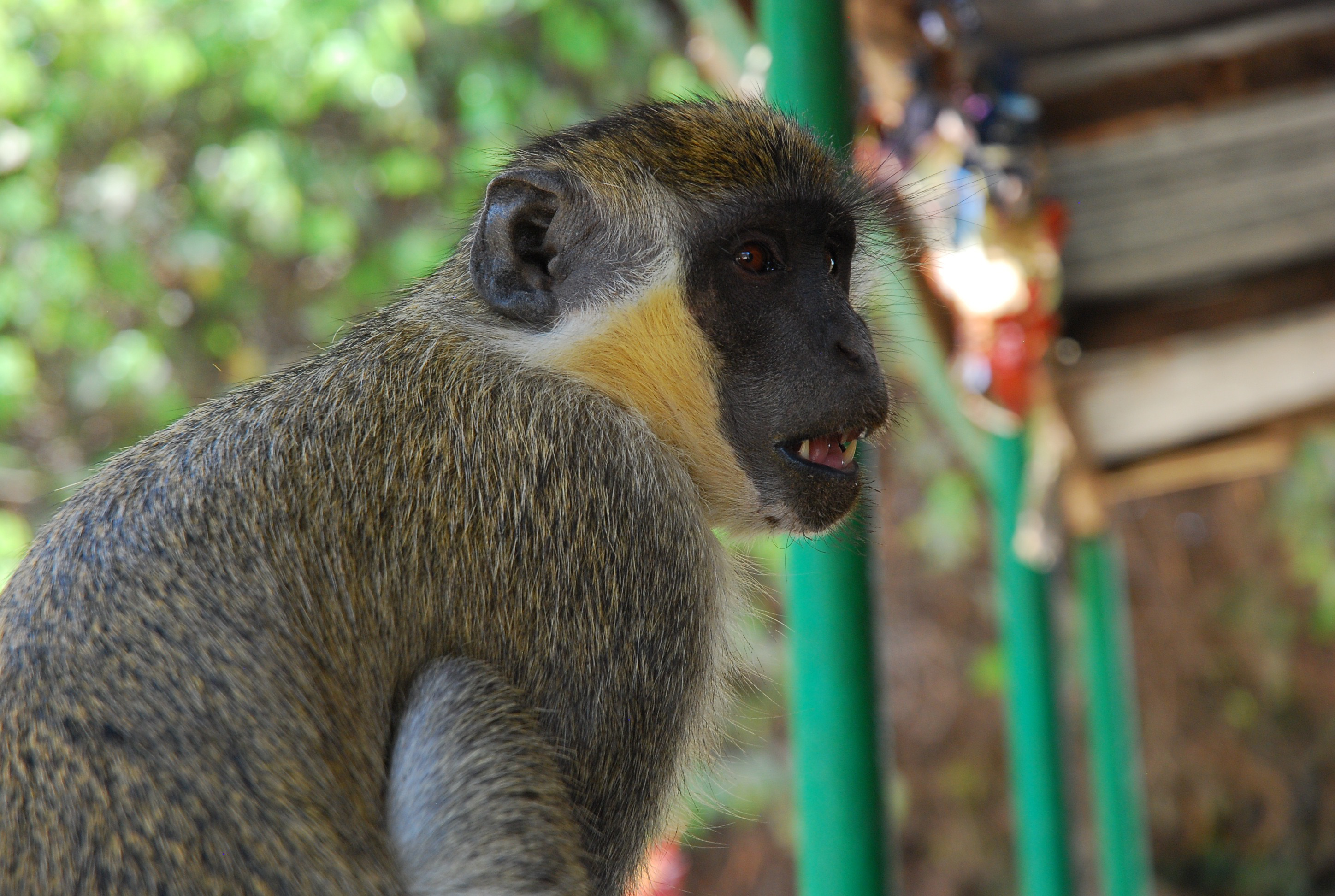

Supplement: S1 Fig — (TIF) [file pone.0235106.s004.tif]
